# Acute pancreatitis and biliary obstruction from metastatic lymph node compression during [^177^Lu] Lu-PSMA-617 therapy: a case report

**DOI:** 10.3389/fonc.2024.1442293

**Published:** 2024-10-07

**Authors:** Gokce Belge Bilgin, Patrick J. Navin, Derek R. Johnson, Oliver Sartor, Ayse Tuba Kendi

**Affiliations:** ^1^ Department of Radiology, Mayo Clinic, Rochester, MN, United States; ^2^ Department of Oncology, Mayo Clinic, Rochester, MN, United States; ^3^ Department of Urology, Mayo Clinic, Rochester, MN, United States

**Keywords:** prostate cancer, Lu-PSMA, theranosctics, acute pancreatitis, inflammation

## Abstract

Radioligand therapies such as [^177^Lu] Lu-PSMA-617 have gained significant momentum in cancer treatment after clinical trials and multicenter studies demonstrated their safety and efficacy. As these innovative treatments become more widespread, rare and unique clinical manifestations are expected to be observed. In this report, we describe a case with metastatic castration-resistant prostate cancer (mCRPC) and peripancreatic lymph node metastases who developed acute pancreatitis following [^177^Lu] Lu-PSMA-617 therapy.

## Introduction

The beta-emitter [^177^Lu] Lu-PSMA-617 showed extended overall survival and a favorable safety profile when added to the standard treatment for patients with mCRPC in the phase 3 VISION trial (1). Following its FDA approval in 2022, it has been established as the standard of care for mCRPC patients who have not responded to androgen-receptor pathway inhibitors (ARPI) and taxane-based chemotherapies.

As the use of [^177^Lu] Lu-PSMA-617 expands, the importance of documenting and sharing real-world experiences, particularly unique and uncommon case presentations, has increased. Therefore, we present a rare case following [^177^Lu] Lu-PSMA-617 radioligand therapy and the strategies used for its diagnosis and treatment.

## Case presentation

A 69-year-old man with mCRPC was referred to our clinic for [^177^Lu] Lu-PSMA-617 therapy following multiple systemic treatments. He was initially diagnosed with metastatic prostate adenocarcinoma (Gleason score of 8; PSA level of 553 ng/mL) with bulky retroperitoneal and peripancreatic lymph node metastases but no bone or solid organ involvement. Despite an initial positive response to hormone therapies, his disease continued to progress, even after undergoing subsequent treatments with immunotherapy (*Sipuleucel-T*), ARPI, and taxane-based chemotherapies. Due to the persistent biochemical and radiologic progression of his disease, he was referred to our clinic for consideration of [^177^Lu] Lu-PSMA-617 therapy.

**Figure 1:**
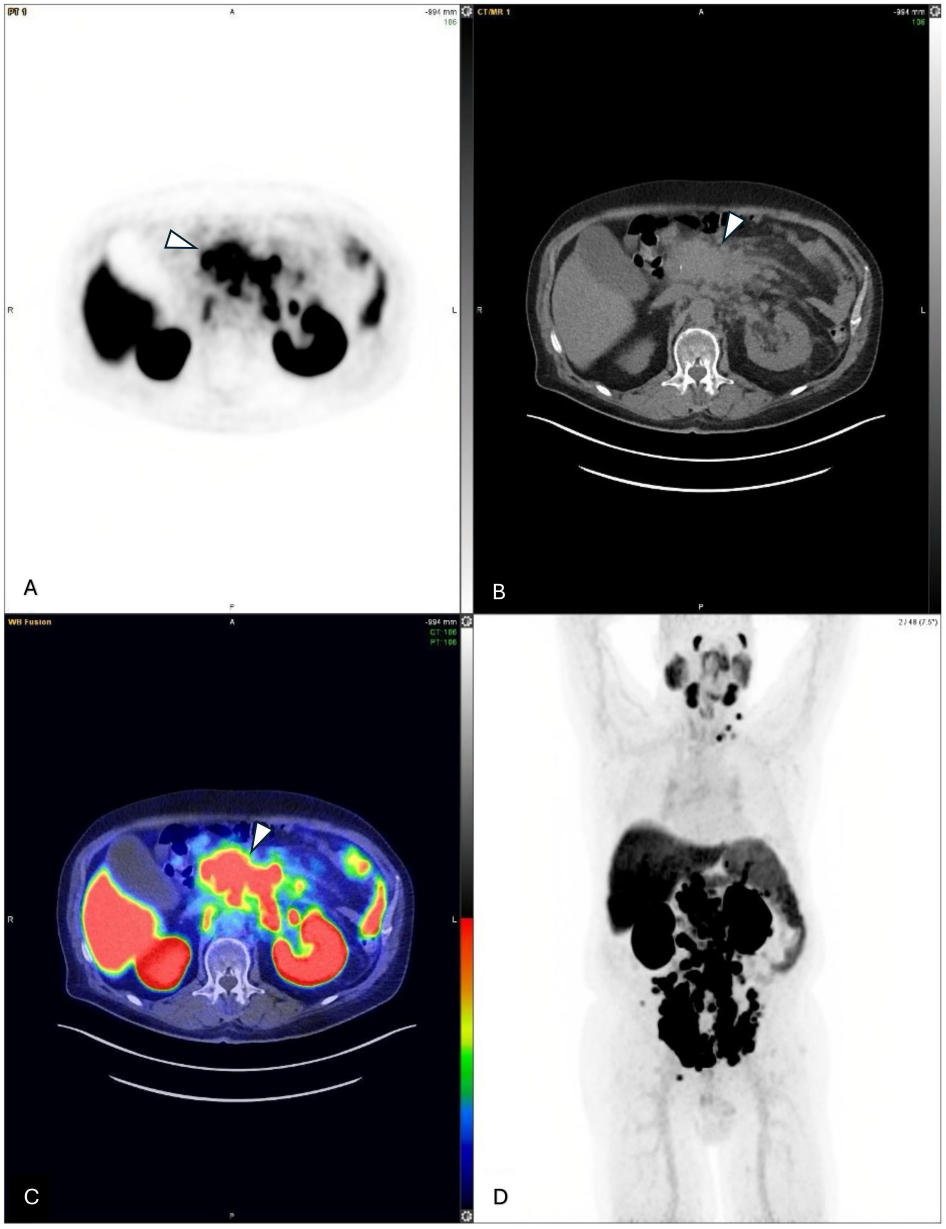
**(A–C)** Axial views of multiple metastatic lymph nodes adjacent to the pancreas from a pre-therapy ^68^Ga-PSMA-PET/CT scan, indicated by arrowheads (molecular imaging PSMA score, 3). **(D)** A pre-therapy ^68^Ga-PSMA PET/CT maximum intensity projection (MIP) image showing multiple PSMA-avid metastatic lymph nodes both below and above the diaphragm.

**Figure 2:**
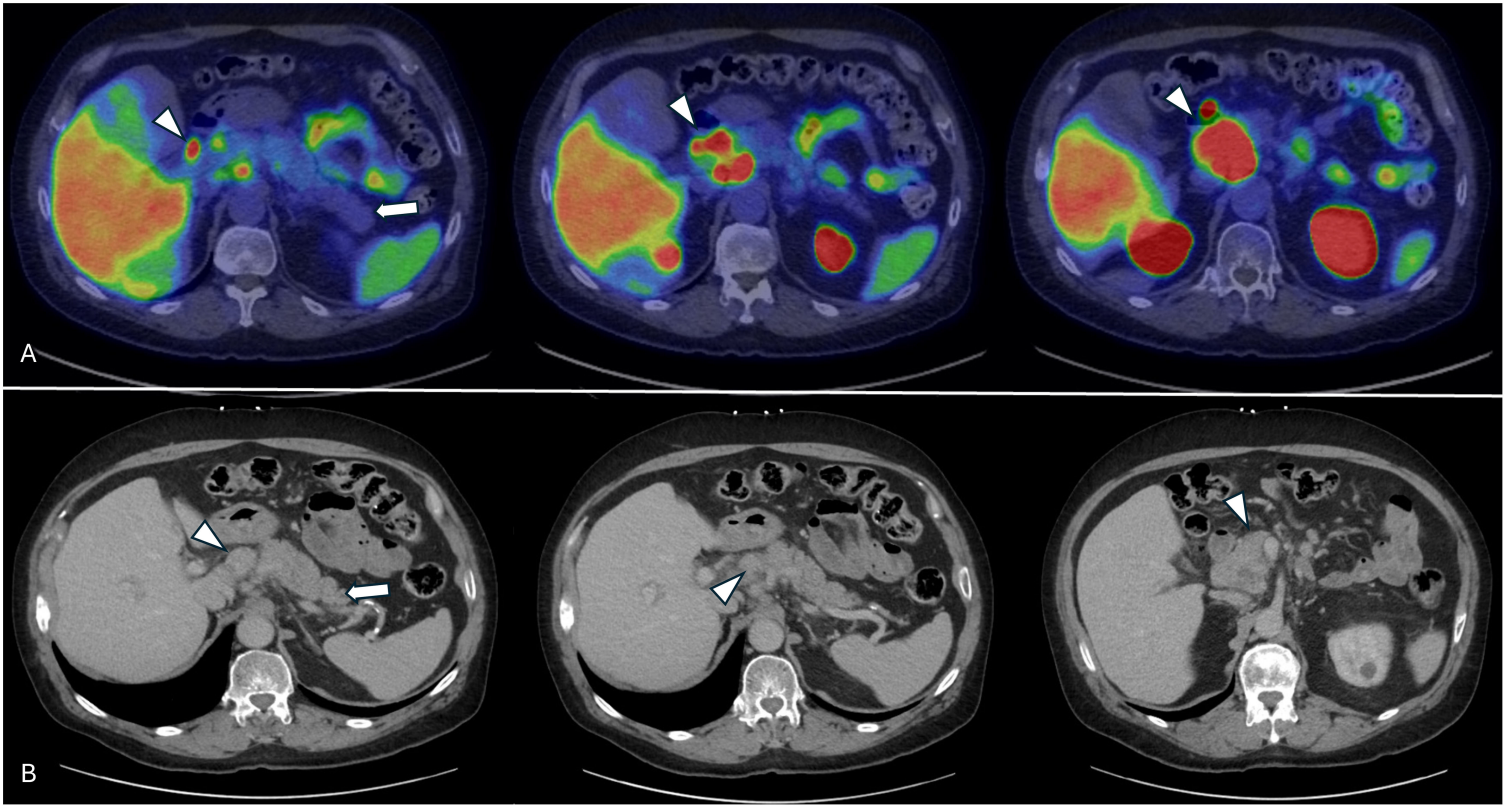
Pretherapy axial views of the pancreas (arrow) and peripancreatic metastatic lymph nodes (arrowheads) showing no inflammatory changes within the pancreas. **(A)** Axial views from the pre-therapy [^68^Ga] Ga-PSMA-11 PET/CT scan. **(B)** Pre-therapy contrast-enhanced CT images showing the pancreas and multiple peripancreatic metastatic lymph nodes.

Pre-therapy therapy [^68^Ga] Ga-PSMA-11 scan confirmed extensive PSMA-avid nodal metastases and identified new peritoneal lesions, with no bone or solid organ involvement (**Figures 1**, **2**). His pre-therapy PSA level was 41.81 U/L. As his blood tests, clinical status, and imaging results were satisfactory, patient was planned for proceeding with the [^177^Lu] Lu-PSMA-617 therapy. While undergoing [^177^Lu] Lu-PSMA-617 treatment, the patient continued his hormone therapy with leuprolide and symptomatic medications, but his chemotherapy and targeted therapies were discontinued.

The first treatment cycle (administered activity: 7.4GBq) was well-tolerated with only decreased appetite, mild nausea, and fatigue. Following the first cycle, his PSA value slightly increased to 48.23 ng/mL. Six weeks later, he presented to the emergency department (ED) with increased nausea and vomiting. During admission, his symptoms were successfully managed with supportive treatment, as laboratory results and an ultrasound showed no abnormalities. He received a second cycle (administered activity: 7.4GBq) without any symptoms. Post-therapy SPECT scan revealed no new metastases or progression in existing lesions. His PSA value was 61.63 ng/mL after the second cycle. Three weeks later, the patient returned to the ED with symptoms of vomiting and back pain. In the patient’s history, there was no record of previous pancreatitis, gallstones, alcohol intake, high blood lipid levels, or other identifiable risk factors for pancreatitis. Blood tests revealed slightly elevated bilirubin and liver enzyme levels. An outside CT scan identified a mass-like lesion adjacent to the pancreatic head, previously noted as PSMA-avid lymph nodes on pre-therapy [^68^Ga] Ga-PSMA-11 PET/CT and contrast-enhanced CT imaging, which was causing biliary duct obstruction and ductal dilation. The obstruction was resolved with an ERCP procedure and common bile duct (CBD) stent placement, leading to symptomatic improvement.

The third cycle (administered activity: 7.4GBq) was completed successfully without any immediate complaints. His post-therapy SPECT/CT scans indicated that the PSMA-avid lymph nodes remained stable in size. Also, his PSA values regressed to 48.09 ng/mL. Three days after the last cycle of treatment, the patient presented to the ED with pain in the lower chest radiating to the mid/upper back. Physical examination revealed abdominal tenderness in the epigastric region. Blood tests showed lipase levels elevated to more than ten times the normal range (1144 U/L). Abdominal CT scan demonstrated significant lymphadenopathy with a patent CBD stent. Meanwhile, an abdominal ultrasound suggested increased pancreatic volume with a significant decrease in echogenicity, possible stent dysfunction, and a hydropic gallbladder with pericholecystic fluid. Gastroenterology recommended continuing supportive care with intravenous fluids and pain medication based on his findings. The patient’s recurrent symptoms were thought to likely be related to radiation-induced peritumoral inflammation and edema in peripancreatic metastatic lymph nodes, which led to compression of the bile duct. As a result, he was prescribed dexamethasone (4 mg twice daily) for inflammation and opioids for pain management. In the following days, the patient was discharged without further complaints.

Due to this development, the patient was evaluated by the tumor board, which supported continuing [^177^Lu] Lu-PSMA-617 therapy. The board recommended dexamethasone to reduce the risk of pancreatitis in subsequent therapy cycles, along with close monitoring of liver function tests and pancreatic enzymes prior to further treatment. Although his abdominal pain and other accompanying symptoms improved, his declining performance status led to a shift in his disease management to palliative care, and [^177^Lu] Lu-PSMA-617 treatment was discontinued.

## Discussion

In our report, we describe a case of biliary obstruction and acute pancreatitis during [^177^Lu] Lu-PSMA-617 therapy from metastatic lymph node compressions. The patient underwent CBD stenting and received anti-inflammatory treatment with steroids among other conservative therapies. His symptoms responded well to these treatments, and the tumor board recommended continuing [^177^Lu] Lu-PSMA-617 with prophylactic steroids. The therapy was discontinued due to the patient’s overall declining condition.

There are several potential causes of acute pancreatitis to consider in our case. The ERCP procedure is commonly associated with mechanical damage to the pancreatic duct or damage from contrast material (2). Studies suggest that complications from ERCP typically arise within first few hours to days after the procedure (2, 3). However, in this case, pancreatitis occurred five weeks post-ERCP, which may decrease its likelihood of being related to the procedure.

Another factor to consider is the progression of cancer and the enlargement of peripancreatic metastatic lymph nodes. However, in our patient, post-therapy SPECT/CT images showed that the PSMA-avid lymph nodes remained stable in size with a decreased radiotracer uptake intensity. Additionally, his PSA values reached a nadir following the third cycle of [^177^Lu] Lu-PSMA-617 therapy. Nevertheless, it is important to note that even subtle changes in the size of these lymph nodes could still cause compression of adjacent structures.

Another potential cause is that radiation-induced inflammation and edema in enlarged PSMA-avid peripancreatic lymph nodes led to bile duct obstruction and subsequent acute pancreatitis. Given that the acute pancreatitis occurred just three days after the third treatment cycle and responded well to steroid treatment, this possibility also warrants consideration. Inflammation is a key response of tissues to ionizing radiation, triggered by vascular damage and necrosis, which promotes leukocyte migration and the release of pro-inflammatory mediators (4, 5). Radioligand therapies exploit these mechanisms to target and eliminate tumor cells, either by causing direct DNA damage or indirectly through the production of free radicals (6). Post-radiation inflammation can manifest in various forms depending on the location of metastatic lesions, potentially causing symptoms such as bone pain, abdominal pain, or temporary structural changes (7–10).

Karfis et al. reported a patient with a rectal neuroendocrine tumor and metastases to the liver, pancreas, and bones, who received [^177^Lu] Lu-DOTATATE as a second-line therapy (11). Two weeks after the first cycle of treatment, their patient experienced acute pancreatitis attributed to inflammation and edema in the pancreatic head metastasis. Similarly, Salner et al. reported a series of patients treated with [^177^Lu] Lu-DOTATATE who exhibited inflammatory reactions within the first week of treatment (8). Of those, one with significant liver metastasis had increased epigastric pain, and two others with extensive mesenteric metastases reported partial small bowel obstruction. These patients were treated with a brief course of oral corticosteroids, a strategy similar to the one we employed in our case.

## Conclusion

[^177^Lu] Lu-PSMA-617 therapy is an established treatment with a favorable safety profile for patients with mCRPC. As demonstrated in the presented case, the therapeutic approach can be customized based on the individual needs of the patient throughout the course of [^177^Lu] Lu-PSMA-617 treatment. Adjunctive anti-inflammatory medications may be used as preventive measures for areas at risk before or during the therapy. However, further research is essential to enhance our understanding and mitigate the effects of such treatments.

## Data Availability

The raw data supporting the conclusions of this article will be made available by the authors, without undue reservation.
